# Application of Quinia in Fever

**Published:** 1876-08

**Authors:** 


Application of Quinia in Fever.—Prof. C. Binz makes (*The Practitioner*, June, 1876,) the following practical remarks on this subject: "It is to be given (1) in large doses from 0.5 to 3.0 grammes (8–48 grains); (2) in a digestible form, that is to say, together with some acid; and (3) during the time when fever tends to decrease.

"The first has been proved by numerous observations in the sick room. Such strong doses are not poisonous, but agree on the whole better with young than with old people. My second rule refers to the fact that in fever the stomach does contain sufficient acid to dissolve the sparingly soluble sulphate. As to the hour of administration, we have to deal here, as a matter of course, with similar conditions as in ague fever. The cause of fever is overcome by quinine most easily when its activity is lowest.

"The complaint has often been raised that quinine so easily causes sickness, and it is generally supposed that the vomiting is produced by direct irritation of the stomach. This is not proved to be the case. On the contrary, if the doses of quinine are continued, at the most only a small part of the second or third are ejected. All later ones remain. The stomach absorbs it as hitherto, the nervous system by degrees tolerates the alkaloid, and now only it begins to act upon the chemical processes of the feverish organism. It is very important not to make use of impure quinine. Quinine containing the less efficacious cinchonine is the most deceptive, for this adulteration is somewhat difficult to detect, at least for many practitioners. Much the same must be said of salicine, the bitter principle of willow."—*Am. Jour. Med. Sciences.*

